# Complete Genome Sequence of* Acinetobacter* sp. Strain NCu2D-2 Isolated from a Mouse

**DOI:** 10.1128/genomeA.01415-16

**Published:** 2017-01-26

**Authors:** Ulrike Blaschke, Gottfried Wilharm

**Affiliations:** Robert Koch Institute, Wernigerode Branch, Wernigerode, Germany

## Abstract

Whole-genome sequencing of *Acinetobacter* sp. strain NCu2D-2, isolated from the trachea of a mouse, revealed the presence of a plasmid of 309,964 bp with little overall similarity to known plasmids and enriched in insertion sequences (ISs) closely related to IS elements known from the nosocomial pathogen *Acinetobacter baumannii*.

## GENOME ANNOUNCEMENT

The genus *Acinetobacter* currently comprises around 50 species with validly published names (1, 2), but it is evident that this number does not yet adequately reflect the actual diversity of the genus (2–4). While some *Acinetobacter* spp. are ubiquitously spread in soil and water habitats, others are nosocomial pathogens such as *A. baumannii* with a propensity to develop resistance to antimicrobial drugs (2, 5). Environmental *Acinetobacter* spp. serve as reservoirs of antibiotic-resistance genes that can disseminate into pathogenic *Acinetobacter* spp. and even into distantly related Gram-negative pathogens (6), illustrating the need for a deeper understanding of the diversity and ecology of the genus.

*Acinetobacter* sp. strain NCu2D-2 was isolated from the trachea of a mouse (*Mus musculus*) captured by a cat in Silstedt, Germany, in 2014, using CHROMagar Acinetobacter (Chromagar, France) as recently described (7). Since partial 16S rRNA and *rpoB* gene sequencing (GenBank accession nos. KM979381 and KM979382, respectively) did not indicate NCu2D-2’s belonging to any described species, whole-genome sequencing was commissioned at GATC (Konstanz, Germany). Genomic DNA was isolated from an overnight culture using a Qiagen Genomic-tip 20/G according to the manufacturer’s recommendations (Qiagen, Hilden, Germany). PacBio RS single-molecule real-time sequencing resulted in 58,959 reads with a total of 818,808,000 sequenced bases and 181.16-fold genome coverage. Genome assembly was performed using the Hierarchical Genome Assembly Process (HGAP version 3).

The genome has a size of 3,048,277 bp and consists of a circular chromosome of 2,738,313 bp and a large circular plasmid of 309,964 bp. NCBI Prokaryotic Genome Annotation Pipeline analysis revealed a total of 2,915 genes, including 2,804 coding sequences and 111 RNA genes, of which 86 define tRNAs and which include seven complete rRNA gene sets as well as four ncRNAs. PacBio modification and motif analysis revealed *N*-6-methylated adenines in motifs TCCAG and WTRAATTYA and an unknown modification within the motif ANNCGGAAGV (modified bases underlined). Motif WTRAATTYA is very similar to WYRAATTYA recently identified in *A. equi* (GenBank accession no. CP012808).

Twelve putative genomic islands were predicted on the chromosome by at least one method applying IslandViewer 3 (http://www.pathogenomics.sfu.ca/islandviewer) (8).

ResFinder version 2.1 (https://cge.cbs.dtu.dk/services/ResFinder) analysis did not reveal acquisition of antimicrobial resistance genes either on the plasmid or on the chromosome (60% identity threshold and 60% minimum length) (9). However, the plasmid harbors arsenic- and tellurite-resistance genes.

The plasmid has a mosaic structure with as many as 19 different insertion sequence (IS) elements predicted by IS Finder (http://www-is.biotoul.fr) (10) with an expect value (E) of 4 × 10^−137^ and below. Some of these IS elements appear in multiple copy number, both on the chromosome and on the plasmid, and many of the IS prototypes, such as ISAba5, ISAba12, ISAba13, ISAba16, ISAba17, ISAba25, and ISAba125, are well known from the nosocomial pathogen *A. baumannii*. These findings illustrate the fluidity of the *Acinetobacter* pangenome and the importance of studying genomes of environmental *Acinetobacter* spp. in order to understand the evolution of the pathogenic species within the genus.

### Accession number(s).

The complete genome of *Acinetobacter* sp. strain NCu2D-2 was deposited in GenBank under the accession numbers CP015594 (chromosome) and CP015595 (plasmid) (Bioproject ID PRJNA315981).
